# Glial Innate Immunity Generated by Non-Aggregated Alpha-Synuclein in Mouse: Differences between Wild-type and Parkinson's Disease-Linked Mutants

**DOI:** 10.1371/journal.pone.0013481

**Published:** 2010-10-26

**Authors:** Cintia Roodveldt, Adahir Labrador-Garrido, Elena Gonzalez-Rey, Rafael Fernandez-Montesinos, Marta Caro, Christian C. Lachaud, Christopher A. Waudby, Mario Delgado, Christopher M. Dobson, David Pozo

**Affiliations:** 1 CABIMER-Andalusian Center for Molecular Biology and Regenerative Medicine, Consejo Superior de Investigaciones Científicos, University of Seville-UPO-Junta de Andalucia, Seville, Spain; 2 Institute of Parasitology and Biomedicine Lopez-Neyra, Consejo Superior de Investigaciones Científicos, Granada, Spain; 3 Department of Chemistry, University of Cambridge, Cambridge, United Kingdom; 4 Department of Structural and Molecular Biology, University College, London, United Kingdom; City of Hope National Medical Center, United States of America

## Abstract

**Background:**

Parkinson's disease (PD) is a progressive neurodegenerative disorder characterized pathologically by the presence in the brain of intracellular protein inclusions highly enriched in aggregated alpha-synuclein (α-Syn). Although it has been established that progression of the disease is accompanied by sustained activation of microglia, the underlying molecules and factors involved in these immune-triggered mechanisms remain largely unexplored. Lately, accumulating evidence has shown the presence of extracellular α-Syn both in its aggregated and monomeric forms in cerebrospinal fluid and blood plasma. However, the effect of extracellular α-Syn on cellular activation and immune mediators, as well as the impact of familial PD-linked α-Syn mutants on this stimulation, are still largely unknown.

**Methods and Findings:**

In this work, we have compared the activation profiles of non-aggregated, extracellular wild-type and PD-linked mutant α-Syn variants on primary glial and microglial cell cultures. After stimulation of cells with α-Syn, we measured the release of Th1- and Th2- type cytokines as well as IP-10/CXCL10, RANTES/CCL5, MCP-1/CCL2 and MIP-1α/CCL3 chemokines. Contrary to what had been observed using cell lines or for the case of aggregated α-Syn, we found strong differences in the immune response generated by wild-type α-Syn and the familial PD mutants (A30P, E46K and A53T).

**Conclusions:**

These findings might contribute to explain the differences in the onset and progression of this highly debilitating disease, which could be of value in the development of rational approaches towards effective control of immune responses that are associated with PD.

## Introduction

Parkinson's disease (PD) is the second most common neurodegenerative disorder, after Alzheimer's disease. It is characterized pathologically by the presence of deposits of aggregated α-synuclein (α-Syn) in intracellular inclusions, known as Lewy bodies, in the *substantia nigra pars compacta* (SN) of the brain [1], [2], and by the loss of dopaminergic neurons [3], [4]. There is considerable evidence indicating a role of α-Syn in the etiology of PD, in which the conversion of α-Syn from soluble monomers to aggregated amyloid-like insoluble forms is a key event in PD pathogenesis [5]. However, the cellular and molecular mechanisms underlying the pathological actions of α-Syn are still not completely understood. Traditionally, α-Syn has been viewed as an exclusively intracellular, cytoplasmic protein which is highly expressed in dopaminergic neuronal cells. Lately, accumulating evidence showing the uptake of extracellular α-Syn by glia and neurons via endocytosis [6], [7], the release and exocytosis of α-Syn to the medium [8], [9], and the presence of α-Syn in cerebrospinal fluid [10], [11] and blood [11] both in its aggregated and non-aggregated forms has pointed at the importance of studying the effects of extracellular α-Syn on surrounding cells in the brain.

Alpha-Syn is a 140-amino acid protein that is highly enriched in presynaptic neuronal terminals, in particular in the neocortex, hippocampus, and SN [12], as well as within astrocytes and oligodendroglia [13], [14]. The physiological role of α-Syn is still being established, but its interaction with pre-synaptic membranes suggests that one function may be the regulation of synaptic vesicle pools, including control of dopamine levels [15]. Alpha-Syn belongs to the group of proteins described as natively unfolded [16], meaning that it does not adopt a well-defined globular structure, but instead a broad ensemble of dynamically interacting and largely disordered conformations [17], [18]. Three missense mutations, A53T, A30P and E46K, as well as multiple copies of wild-type (Wt) α-Syn, are linked to hereditary, early-onset PD [19]–[22]. *In vitro* studies have shown that the ensemble of α-Syn conformers is perturbed by the mutations [23], at least in the cases of A30P and A53T studied. Presumably as a result of the differences in their structural, biophysical and biochemical characteristics, the various mutants have been reported to have different cytotoxic effects, and this cytotoxicity to be mediated by different pathways (reviewed in [24]). Nevertheless, the factors contributing to both familial and sporadic cases of PD are not understood in any detail.

Even though the central nervous system (CNS) has been traditionally seen as an immune-privileged organ, it has become increasingly evident that inflammation is actively involved in the pathogenesis of various degenerative diseases including multiple sclerosis, Alzheimer's disease and PD (reviewed in [25]). Indeed, accumulating evidence indicates that the onset and progression of PD is accompanied by a robust and highly localized inflammatory response mediated by reactive astrocytes and activated microglia in affected areas in the brain of PD patients [26]–[30]. Whether microglial activation protects or exacerbates neuronal loss is currently the subject of debate [31]–[34]. Significantly, a link was established a few years ago between extracellular, aggregated α-Syn and activation of microglia [35] leading to dopaminergic neurotoxicity, and a few recent *in vivo* studies have shown that microglial activation and neurodegeneration can be directly caused by α-Syn overexpression [36]–[40]. Even though evidence has accumulated pointing at the importance of the immunological features of α-Syn related to the pathogenesis of Parkinson's disease (reviewed in [2], [41]), the effects of extracellular α-Syn on the cellular and molecular components of the immune system linked to PD pathology [42], remain largely unexplored.

Up to this point, research on α-Syn-mediated cell response has focused primarily on the effects of aggregated α-Syn on neuroinflammation [43] or on activation of microglia [24], [35], [44]–[46]. In turn, most of these studies have focused on nitrated α-Syn [43], [45], [46], assuming that extracellular α-Syn has been modified in a similar manner to α-Syn found in Lewy bodies [47], [48] −a typically pro-oxidative environment− an assumption that is still uncertain [42] and might not be valid for secreted α-Syn. Moreover, it has been recently shown that non-aggregated, exogenous α-Syn can regulate the key brain cytoactive molecules matrix metalloproteinase-9 and tissue plasminogen activator in glial cells [49], [50], and induces higher TNF-α, IL-1β and ROS release levels than aggregated α-Syn in microglia [50]. Furthermore, it has also been observed that, in contrast to the aggregated form, monomeric α-Syn enhances microglial phagocytosis [51]. These results and other recent findings point at the importance of exploring the effects on the immune response of non-aggregated/monomeric as well as aggregated extracellular α-Syn. Despite the fact that some investigations in this direction have been done using monocytic cell lines [52], human astrocytes [53], or microglia [37], nothing has been reported about the cytokine expression profile of primary microglial cells induced by non-aggregated α-Syn, under conditions where the aggregation state of the protein has been characterized. Likewise, apart from a recent article focusing on the pro-inflammatory effects of an α-Syn double mutant which does not exist in nature [54], a comparative study of wild-type α-Syn and pathologically relevant α-Syn mutants is still lacking. Moreover, so far there are no data available on key chemokines that might control the differential homing and activation of T cell subsets, monocytes and glial cells in this context.

In this work, we have compared the activation profile of non-aggregated, extracellular Wt α-Syn and its PD-linked variants, by measuring the release of key interleukins and chemokines in glial cells. Our findings demonstrate significant differences in the immune response profiling of Wt α-Syn and PD-related mutants that might indicate the existence of different pathways towards PD onset and progression.

## Results and Discussion

### Characterization of α-Synuclein preparations

**Figure 1:**
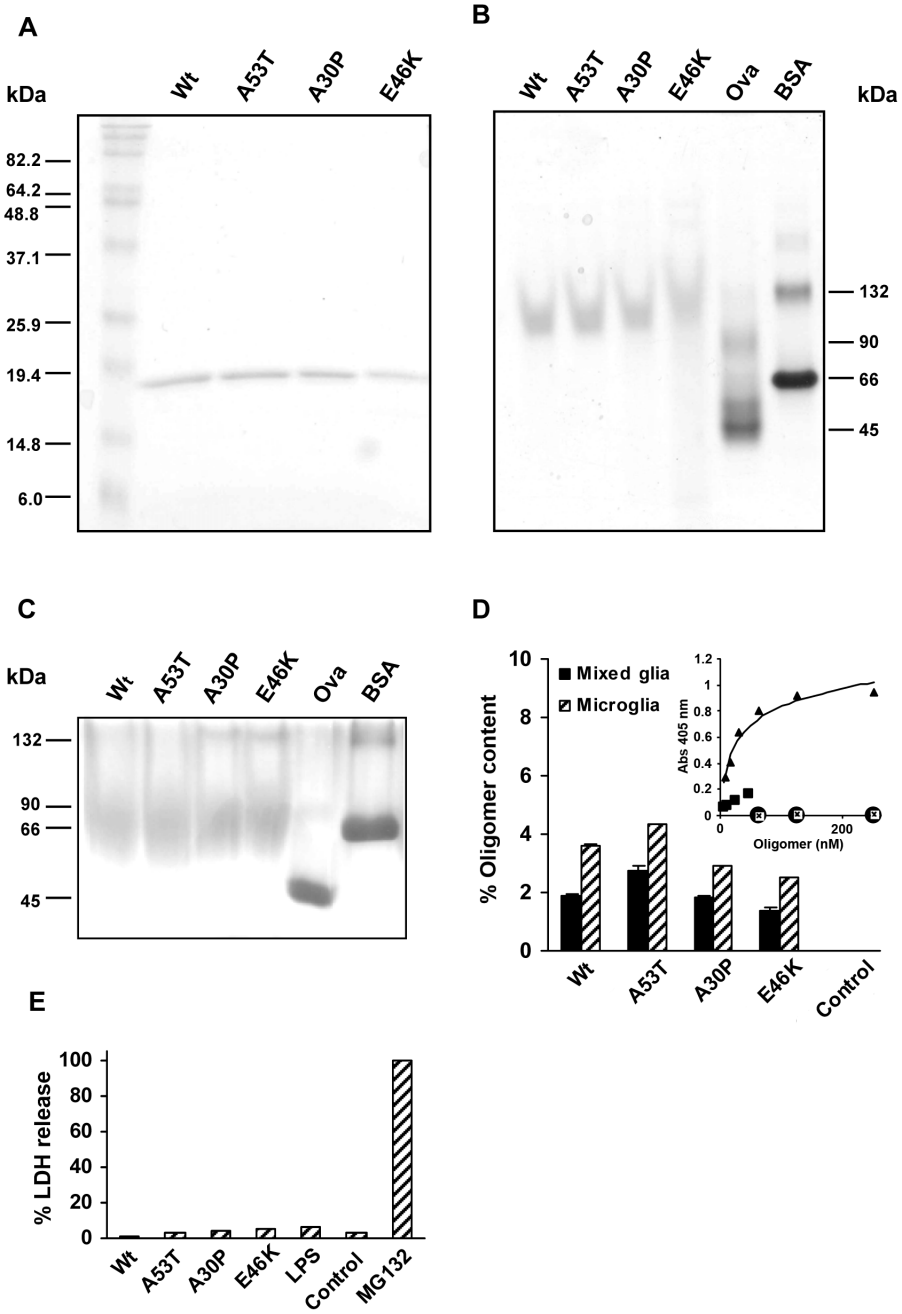
Determination of the purity, oligomeric state and cytotoxic effects of preparations of α-synucleins. (**A**) SDS-PAGE electrophoresis and (**B**) native PAGE electrophoresis of wild type (Wt) and mutant (A53T, A30P and E46K) α-Syn preparations; (**C**) BN-PAGE of wild type (Wt) and mutant (A53T, A30P and E46K) α-Syn preparations; (**D**) α-Syn oligomer content after 20 hr incubation with cells (on a protomer basis), relative to the initial amount of exogenously added α-Syn, as determined by sandwich ELISA. (**E**) LDH release in mixed glial cultures following incubation for 20 hours with the highest concentration of α-Syn used in our experiments, i.e. 5 µg/ml. MG132, a proteasome inhibitor, was used at 6 µM as a control. Values are means from triplicate measurements.

It is known that α-Syn has a tendency to self-assemble under certain conditions to form dimers [55], [56] and higher order oligomeric species in addition to amyloid-like fibrils [57]. In order to assess the purity and oligomerization state of the α-Syn preparations used in this study, we subjected the purified Wt and mutant α-Syn protein variants to electrophoretic analysis (**Figure 1**). As expected from additional analysis by mass spectrometry (not shown), each of the four α-Syn preparations migrated as a single, well defined band corresponding to ca. 14.5 kDa as analysed by SDS-PAGE (**Figure 1A**). However, when the samples were subjected to native PAGE (**Figure 1B**), they migrated as less defined bands of ca. 110–120 kDa, as found previously for monomeric α-Syn a natively unfolded protein with a large charge/mass ratio under these conditions [58]. Finally, the rather smeared bands corresponding to ca. 50–60 kDa observed by Blue Native PAGE (BN-PAGE) (**Figure 1C**), in which proteins migrate solely as a function of their apparent mass [59], indicates that the α-Syn preparations are monomeric. Taken together, our data demonstrate a well defined monomeric state for the functional characterization of immune-elicited reponses by the α-Syn protein variants. In order to analyse the oligomeric state of α-Syn present in the cell cultures after 20 hours, a specific and sensitive ELISA assay [60] was used. As can be observed, a low amount of α-Syn oligomers were formed by the end of the incubation step with cells, with ≤4.3% of oligomers relative to the initially added α-Syn (**Figure 1D**). In addition, in order to assess the total amount of α-Syn still present in the medium by the end of the incubation step with cells, a time-course quantification of α-Syn in the culture supernatants was performed after 1, 6 and 20 hours (**Figure S1**). We found that after 20 hours, the α-Syn content was still ca. 60% of the exogenously added amount.

In order to assess the level of cytotoxicity induced by α-Syn at the concentrations used in this study, we next performed lactate dehydrogenase (LDH) release assays with microglial cells. After incubating primary mixed cultures for 20 hours with the α-Syn variants at 5 µg/ml (the highest concentrations used in this work), the cytotoxicity levels displayed by all four α-Syn variants were found to be very low (≤8%) and similar to basal control levels (**Figure 1E**). Therefore, it can be concluded that the parameters measured in this study after α-Syn treatment of cell cultures are not linked to alterations in cellular viability and therefore represent a specific α-Syn immune mediated-response.

### Characterization of primary microglial cultures

**Figure 2:**
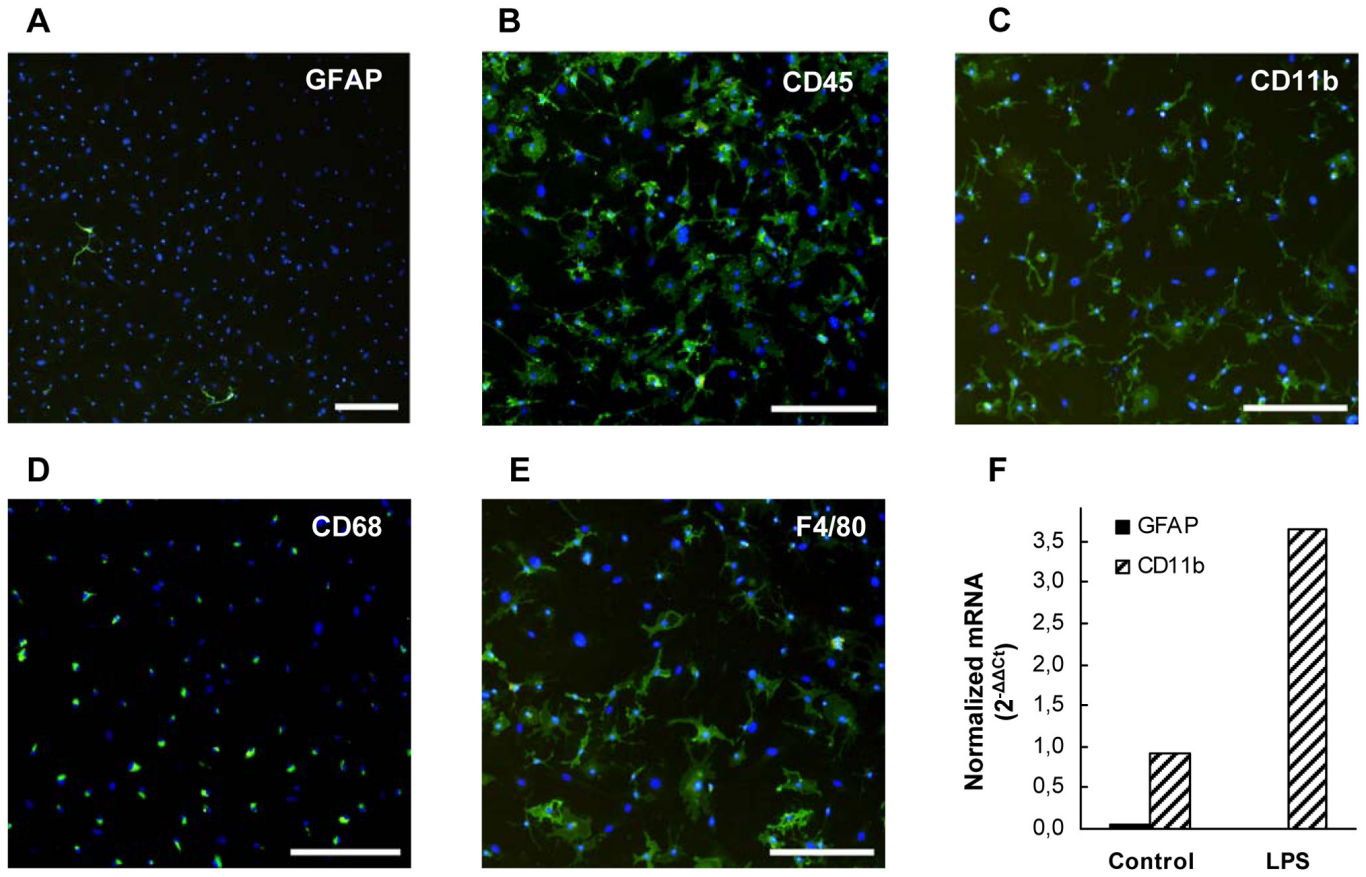
Evaluation of the purity of isolated microglial cell fractions. Immunofluorescence characterization of purified microglial cell cultures for the specific macroglial lineage marker GFAP (**A**), the pan haematopoietic lineage marker CD45 (**B**), and the mature macrophages markers CD11b (**C**), CD68 (**D**), and F4/80 (**E**). Nuclei are counterstained in blue with Hoechst 33342. Scale bar: 100 µm. mRNA expression (qRT-PCR) from isolated microglial preparations after no stimulation (control) or after stimulation with 1 µg/ml LPS for 20 hours (**F**). Specific primers for amplifying the GFAP and CD11b genes were used.

Microglial cells were purified from long-term cultures of neonatal mouse brains as described in the Materials and Methods section. The purity of the isolated microglial fraction was evaluated by two independent approaches. First, immunofluorescence procedures were used as shown in **Figure 2**, where the absence of contaminating macroglial cells after purification was confirmed since less than 1% of the total cells stained positively for GFAP, an intermediate filament specifically expressed in macroglial cells (**Figure 2A**). As a positive control, most of the cells detached by trypsinization of the long-term cultures from neonatal mouse brains –expected to be macroglia– were found to be strongly immunostained for GFAP (not shown). As a second measure of the purity of microglial cell cultures, the majority of cells were found to express the pan haematopoietic lineage marker CD45 (**Figure 2B**), the monocyte-macrophage marker CD11b (**Figure 2C**), and the mature macrophage markers CD68 and F4/80 (**Figures 2D** and **2E**, respectively). In addition, quantitative RT-PCR was employed to amplify specifically the GFAP and CD11b genes (**Figure 2F**), and the results indicate no detectable mRNA expression of the GFAP macroglial marker, and a clear up-regulation of the microglia-specific CD11b gene expression after stimulation by lipopolyssacharide (LPS). Taken together, the cell marker profiles observed confirms the high purity of the microglial cultures and hence to the reliability of the results obtained using our purified microglial cell culture model.

### Pro-inflammatory response of α-Syn-stimulated glia and microglia

In the normal CNS, brain tissue provides an immunosuppressive environment, which seems to be important for the proper function of the CNS. Under circumstances that cause disruption of this environment, including chronic inflammatory conditions in neurodegenerative diseases, a variety of immune regulatory and inflammatory mediators can be activated [25]. Although various types of cells have been identified as sources of cytokines in the CNS, microglia appear to be a principal source of pro-inflammatory and immune regulatory cytokines [25], [61]. In order to explore the immunological properties of extracellular, non-aggregated α-Syn, as well as to evaluate the importance of the cellular context in this process, we measured by ELISA the release of a series of key cytokines after incubation for 20 hours of mouse primary cultures of mixed glia and isolated microglia, with exogenously added Wt α-Syn or the early onset PD-linked α-Syn variants A30P, E46K, and A53T (at 0.2, 1 or 5 µg/ml).

**Figure 3:**
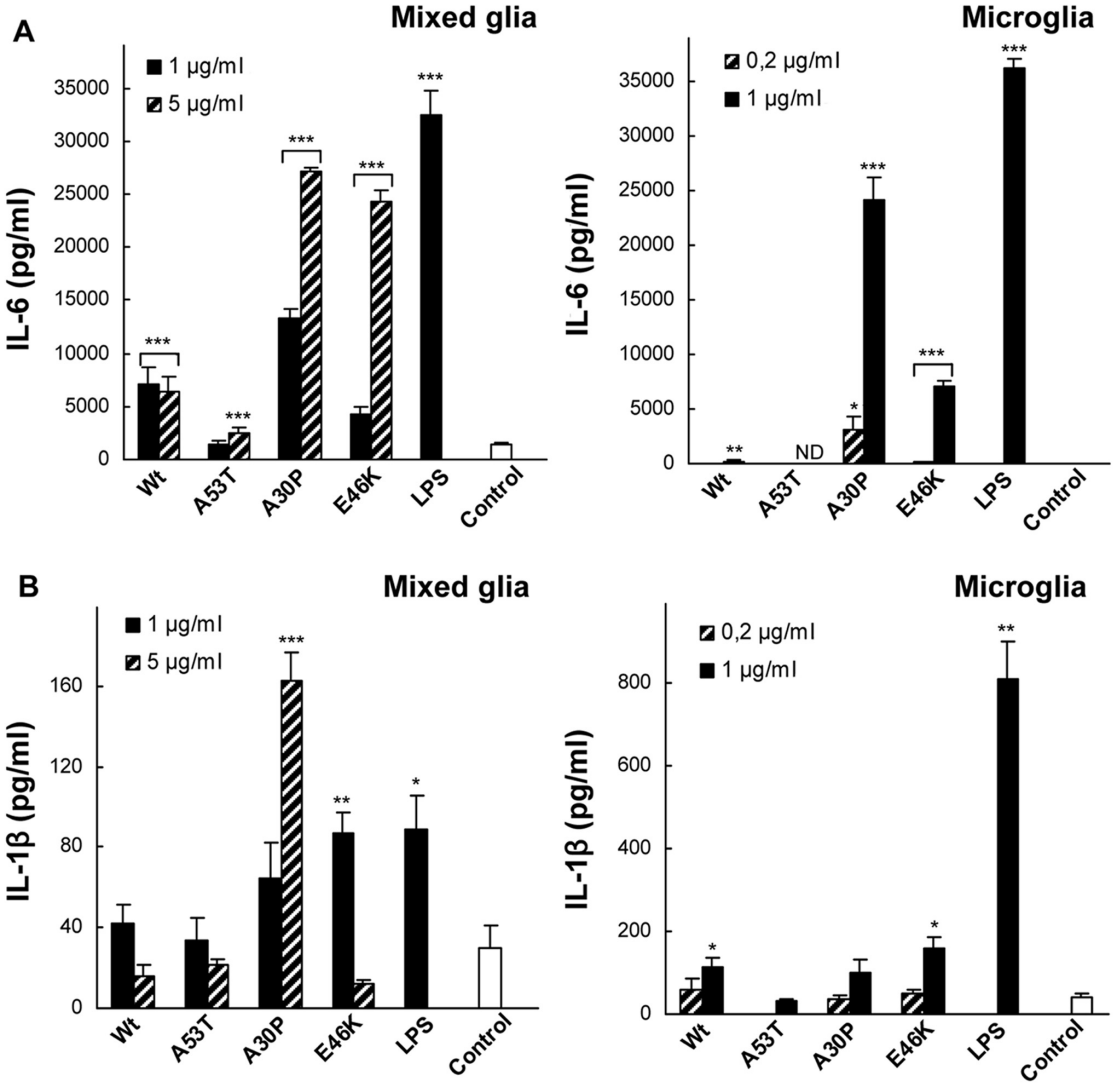
Pro-inflammatory interleukin profile of α-Syn-stimulated primary mixed glial and isolated microglial cultures. IL-6 (**A**) and IL-1β (**B**) release was measured by ELISA in culture supernantants of mixed glia (left) and microglia (right) after a 20-hour treatment with monomeric Wt or mutant α-Syn variants or lipopolysaccharide (LPS). Values are mean ± S.E.M. (n = 4). * P<0.05, ** P<0.01, *** P<0.001. The results shown are representative of two or three independent experiments with microglia and mixed glial cultures, respectively.

First, we assayed the release of IL-6, TNF-α, IFN-γ and IL-1β, four key pro-inflammatory cytokines (**Figure 3** and **S2**). Wild-type α-Syn moderately stimulated the release of IL-6 in mixed glial cultures (**Figure 3A,** left panel), but contrary to findings for aggregated Wt α-Syn [62], this effect was hardly detectable on isolated microglia (**Figure 3A,** right panel). And in contrast to the comparable IL-6 response observed for the four α-Syn variants reported in a study using the human U-373 MG astrocytoma cell line [53], we found remarkable differences in their behaviour, notably a very strong IL-6-mediated pro-inflammatory response induced by the A30P and E46K variants after stimulation of both mixed glial and microglial cultures. These differences could be explained by the nature of the cell line used by Klegeris and coworkers. Alternatively, given that astrocytes are the most abundant glial cell population of the CNS participating in local innate immune responses; this result could be consistent with our finding of less marked differences (**Figure 3A**) between the α-Syn variants in mixed glial cultures. The A53T variant, however, caused a weak but significant increase in IL-6 levels on total glia but not on isolated microglia, similar to Wt α-Syn but even less prominent. In the case of TNF-α and IFN-γ levels measured in microglial cultures, only stimulation with A30P produced a significant increase (**Figure S2**), partially coinciding with the IL-6 observed profile. Therefore, A30P and E46K appear to drive IL-6, TNF-α, and IFN-γ cytokine secretion in the context of PD-affected glia.

When we assayed the levels of IL-1β from mixed glial cultures (**Figure 3B,** left panel), only the A30P and E46K α-Syn variants showed a significant stimulatory effect. Interestingly, when tested on isolated microglial cells, while A30P did not cause a significant rise in IL-1β levels relative to the control, Wt α-Syn −in agreement with a previous report of mRNA levels in a comparable experiment design [37]− and especially E46K, showed a very significant increase in cytokine release (**Figure 3B,** right panel). Taken together, these results suggest that A30P and E46K, as opposed to either Wt α-Syn or the A53T variant, induce a pro-inflammatory response in glia. Remarkably, only two of the three PD-linked variants of α-synuclein were IL-1β inducers in primary glia, and contrary to expectations from previous studies performed using cell line cultures and other studies involving modified α-synuclein species, our results show that the A53T α-Syn variant has instead a very modest activity in regulating the innate immune response by primary glia and microglia. These findings indicate the need for further detailed studies on the role of the glia in early PD onset before detailed conclusions as to the role of the latter in PD can be drawn.

Previously, the available data had shown that the A30P and A53T α-Syn variants, but not the Wt and the E46K forms, efficiently induced the release of IL-1β when added to THP-1 macrophage cell line cultures [52] and that all the α-Syn variants were able to increase IL-1β secretion in THP-1 cells only when co-treatments with INF-γ were included, suggesting a pro-inflammatory response in already immune-primed THP-1 cells [52]. Our data, obtained with primary microglial cultures, indicate a very different behaviour. Thus, Wt and E46K α-Syn in our study appear to be pro-inflammatory in primary microglia, while the A30P and A53T variants seem to be unable to produce a significant response. These differential responses of these two studies point at the importance of the differentiation/maturation status of the primary microglia, which suggests that there could be multiple subtle but important differences in immune responses during the establishment of PD. It is interesting that these differences were only revealed in primary settings that were not over-primed, as was the case of IFN-γ treated THP-1 cells.

The observed effects on IL-1β secretion by primary glia and microglia are of particular interest considering the emerging role of an adaptive immune response in PD, in particular by CD4^+^ T cells [63]. Given IL-1's known effects to promote T cells responses, our findings on IL-1β regulation by α-Syn variants in innate immunocompetent cells require further attention in view of the potential effects of the latter in mediating immune tolerance and T effector responses.

### IL-10 regulation by α-Syn-stimulated glia and microglia

**Figure 4:**
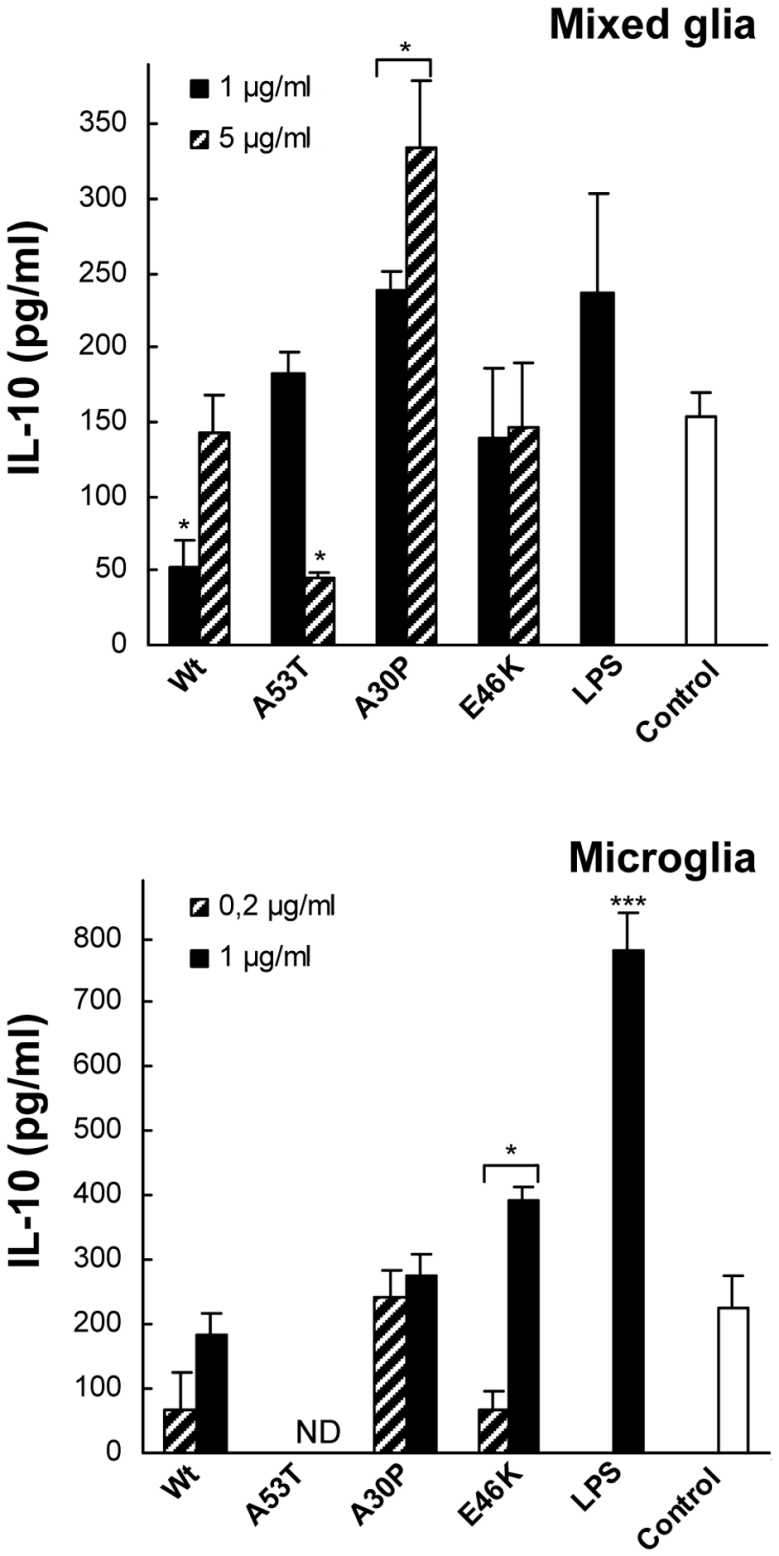
Immunoregulatory effect of α-Syn-stimulation in primary mixed glial and isolated microglial cultures. IL-10 release was measured by ELISA in supernatants of α-Syn-stimulated mixed-glial cultures (top) and microglia (bottom) after a 20-hour treatment with monomeric Wt or mutant α-Syn variants, or lipopolysaccharide (LPS). Values are mean ± S.E.M. (n = 4). * P<0.05, ** P<0.01, *** P<0.001. The results shown are representative of two or three independent experiments with microglia and mixed glial cultures, respectively.

Although most studies in the past have focused on microglial production of pro-inflammatory cytokines, a large body of evidence has supported the notion that microglia also produce cytokines with anti-inflammatory or regulatory activities [25]. Indeed, a strong induction of IL-10 −recognized as an anti-inflammatory cytokine− had been observed for microglial cells stimulated with nitrated, aggregated Wt α-Syn [62]. We therefore investigated the effects of non-aggregated and unmodified α-Syn on glial secretion of IL-10 −a Th2 immunoregulator− which reduces cytokine production by Th1 cells (**Figure 4**). Our results show that only the A30P variant produced a significant increase in IL-10 levels in mixed glial cells (top panel) whilst, on the contrary, the A53T variant caused a significant reduction of IL-10 basal levels, also observed in microglia, likely suggesting a lack of microglial response, a differential uptake by microglia, or an effect of the uptaken α-Syn on the endogenous IL-10 when A53T is present. In this sense, it has been reported a link between α-Syn and the microglial activation features [64], including phagocytic ability.

In microglial cells, on the other hand, only the E46K variant produced an increase in IL-10 levels as compared to the control (bottom panel). These results might suggest that microglial cells, in order to produce α-Syn-driven endogenous IL-10, require both IL-6 and IL-1β secretion. In this sense, while Wt α-Syn increased only IL-1β production in microglial cells, A30P increased only the IL-6 production. These facts could reflect the requirement of a doubly activated state of the microglia for IL-10 production. Although the general mechanism that generate IL-10 production within the CNS during neuroinflammation is still not well enough understood, our results support a role for α-Syn in the modulation of the microglial phenotype as suggested by Austin and collaborators [64].

### Chemokine release profiles by α-Syn-stimulated glia and microglia

**Figure 5:**
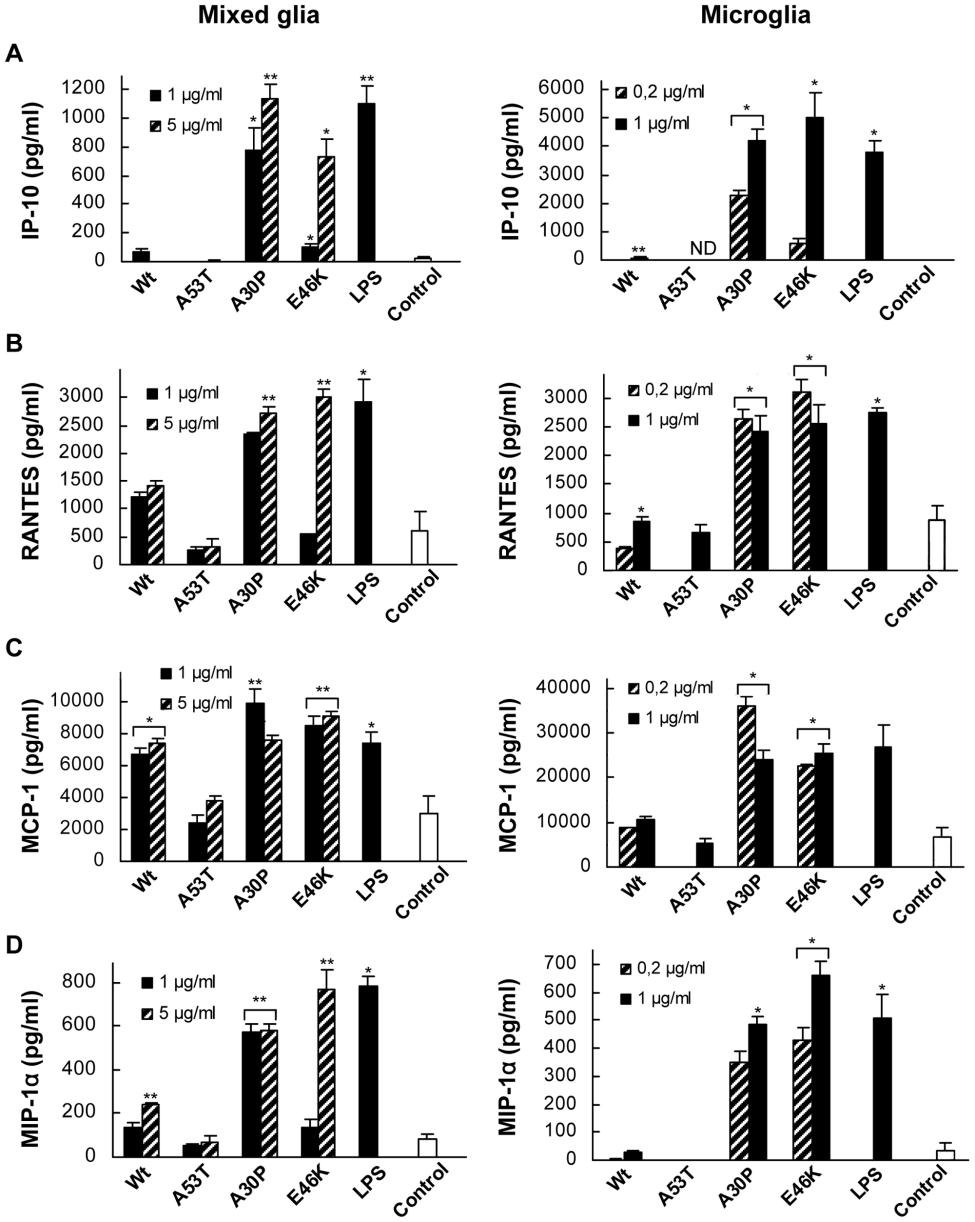
Chemokine release profile of α-Syn-stimulated primary mixed glial cultures and isolated microglia cultures. IP-10 (**A**), RANTES (**B**), MCP-1 (**C**), and MIP-1α (**D**) were measured in supernantants of mixed-glial cultures (left) and microglia (right) after a 20-hour treatment with monomeric Wt or mutant α-Syn variants, or lipopolysaccharide (LPS). All chemokines were assayed by ELISA as described in Materials and Methods. Values are mean ± S.E.M. (n = 2). * P<0.05, ** P<0.01. The results shown are representative of two and three independent experiments with microglia and mixed glial cultures, respectively.

Chemokines are involved in a wide variety of disorders in the CNS and their actions contribute to reactive glial changes and neuronal injury in neuroinflammatory conditions [65]–[67]. We therefore sought to determine the chemokine release profiles of glial and microglial cells induced by non-aggregated Wt and PD-linked α-Syn variants. In particular, we assayed the release of IP-10/CXCL10, RANTES/CCL5, MCP-1/CCL2 and MIP-1α/CCL3 chemokines (**Figure 5**).

The regulation by α-Syn of IP-10/CXCL10 in CNS cells has not been previously reported. In addition, the only data available regarding RANTES/CCL5 levels in PD are from a transgenic rat model for A53T α-Syn [38], or from sera and thus relate to peripheral dysregulation in the cytokine network associated with PD patients [68]–[70], and although no information is given on the genetic background of these PD patients, these studies reported increased levels of circulating RANTES/CCL5. Our results show large and comparable effects for A30P and E46K variants on IP-10/CXCL10 and RANTES/CCL5 release, both by mixed glial cultures and by microglia (**Figures 5A** and **5B**). Remarkably, neither Wt nor A53T in both glial cultures and microglia produced a relevant increase in IP-10/CXCL10 or RANTES/CCL5 secretion, the latter result in agreement with the one reported for the striatum and SN in the (A53T) rat model [38]. These data suggest a specific role for A30P and E46K α-Syn variants associated with enhancement of Th1-cell recruitment, activation, and effector potential.

Besides their role as chemoattractants, IP-10/CXCL10 and RANTES/CCL5 are known to induce T-cell proliferation and cytokine production [71], suggesting a role for A30P and E46K α-Syn variants beyond purely innate pro-inflammatory responses. In this sense, IP-10/CXCL10 is pivotal in generating antigen-specific T-cells [71]. As the possible roles of adaptive immune responses in PD is gaining increasing attention [72], [73], our data should contribute to a better understanding of the differential immune responses exerted by the different α-Syn variants in terms of augmented microglia and macrophage recruitment and/or activation. The strong pro-inflammatory species (IL-1β, IL-6, IP-10/CXCL10, and RANTES/CCL5) whose release the A30P and E46K variants seem to promote in the CNS could lead to augmented macrophage recruitment and/or activation. This view is supported by the observation of upregulation of MCP-1/CCL2 and MIP-1α/CCL3 by A30P and E46K (**Figures 5C** and **5D**). Interestingly, the large effects observed for A30P and E46K were much more pronounced in microglia, indicating a context-dependent stimulation mechanism. Further studies to asses the extent to which peripheral inflammation may amplify the neuroinflammation contributing to PD are needed, but our results raise the possibility of the need for a more personalised manipulation of the different PD situations in terms of immunosupressory treatments and/or immunomodulatory therapeutic approaches.

Previously, in addition to increased levels of TNF-α, IL-6, and INF-γ, stimulation of microglia with nitrated, aggregated α-Syn had been shown to enhance the secretion of MCP-1/CCL2 [46], [62]. In this work, we also found significantly higher levels of MCP-1/CCL2 induced by non-aggregated Wt α-Syn, as well as a similar MCP-1 release profile in mixed glial cultures for Wt α-Syn and its A30P and E46K variants, while A53T caused no detectable chemokine release (**Figure 5C**).

In summary, stimulation of total glia and microglia with A30P and E46K variants show large increases of IP-10/CXCL10, RANTES/CCL5, MCP-1/CCL2 and MIP-1α/CCL3 levels. Wt α-Syn, however, only induced the release of MCP-1/CCL2 and MIP-1α/CCL3, but not in isolated microglia, suggesting potential differential effects of Wt α-Syn and its variants on Th1/Th2 activation and/or recruitment, and a more prominent effect for A30P and E46K α-Syn variants in adaptive immune responses in PD.

### Effect of non-aggregated α-Syn stimulation on microglial phagocytosis

**Figure 6:**
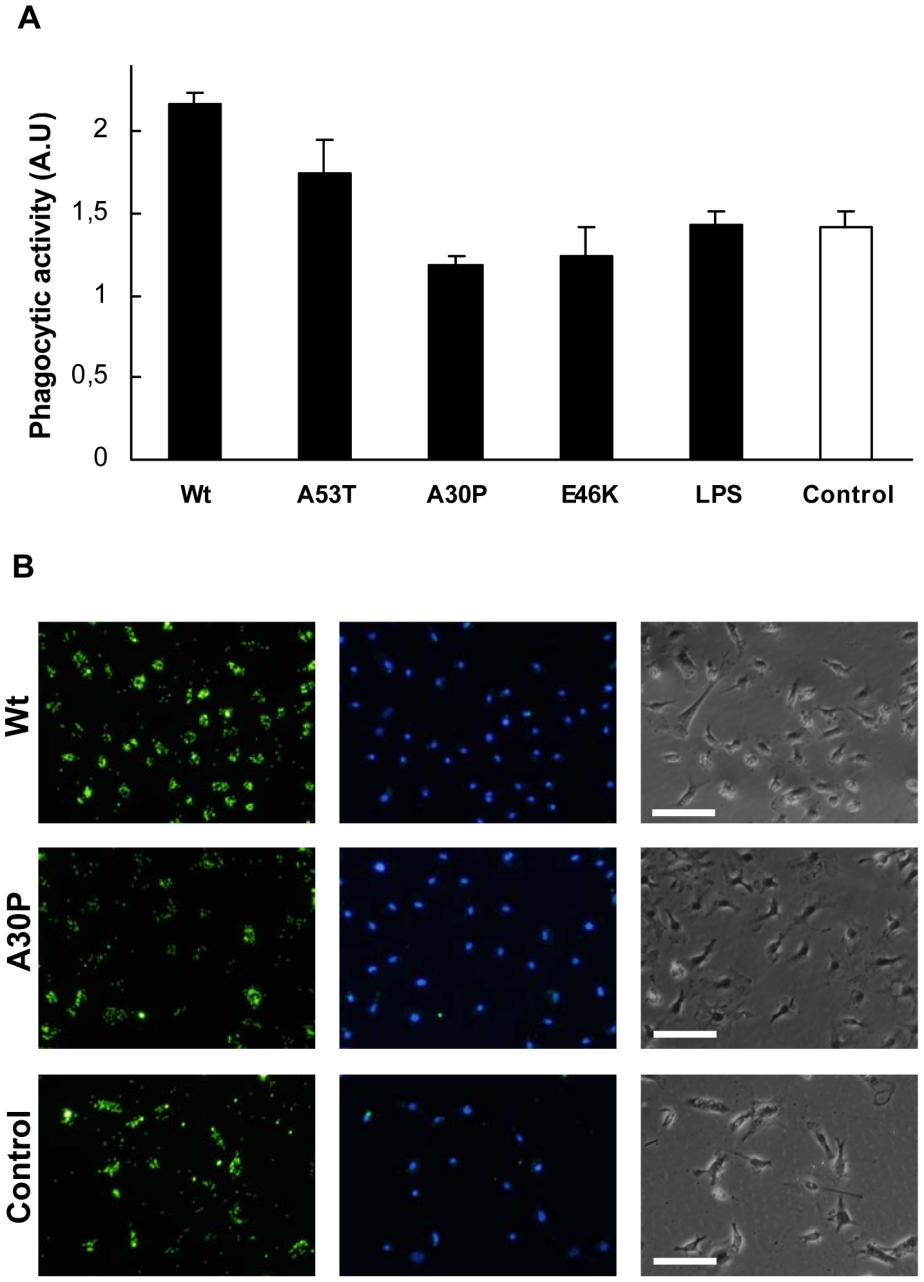
Effect of α-Syn-stimulation on microglial phagocytosis. (**A**) After treatment of the primary microglial cell cultures with α-Syn (1 µg/ml) for 20 hours, cells were incubated with fluorescent microspheres for 1 hour. After fixing the cells, phagocytosis was assessed by fluorescence microscopy analysis. The phagocytic index was calculated by dividing the fluorescence from the phagocytosed microspheres by the total number of cells in the images. Four images were analysed for each sample in each experiment, and the results shown are representative of three independent experiments. A.U.: arbitrary units. (Representative microscopy images used to determine the phagocityc activity of microglial cultures stimulated with Wt (top) an A30P (centre) α-Syn, or non stimulated microglial cells (bottom). From left to right, green fluorescent microspheres, Hoechst-stained cells, and cells as observed in the absence of fluorescence. Scale bar: 50 µM.

Phagocytosis is believed to be involved in steady-state tissue homeostasis, via the clearance of apoptotic cells, and the promotion of tissue repair and resolution of the wound [66], [74]. These aspects are related to the ‘alternative activation’ and acquired deactivation of the microglia, as a counter phenotype to the ‘classically activated’, pro-inflammatory, microglia [74]. Therefore, in order to assess the role of Wt α-Syn and the PD-linked variants on phagocytosis, we used fluorescein-conjugated tracker microparticles for measuring the phagocytosis capacity of differentially activated primary microglial cells (**Figure 6**).

It has been previously reported that monomeric Wt α-Syn enhances phagocytosis of a microglial cell line [51], and indeed, our results show that Wt and A53T α-Syn promote phagocytosis of microglial cells (**Figure 6B**), while the opposite effect was observed for cell cultures stimulated by the A30P and E46K α-Syn variants (**Figures 6A** and **6B**). Interestingly, the phagocytic capacity measured for the stimulated microglia was observed to be inversely correlated to the IL-6 release levels induced by the different α-Syn variants (**Figure 3A**). Our findings point to induction of differential microglial phenotypes by α-Syn variants. Thus, A53T α-Syn, which was not associated with a robust pro-inflammatory activity, produced an increase in the phagocytic capacity that could reflect an alternative activation state of the microglia, as observed with CNS damage [61]. However, Wt α-Syn, which was associated with a moderate proinflammatory response in our model, promoted phagocytosis in microglia, indicating a combination of alternative and classical activation states, a scenario that has been related to chronic inflammatory processes such as those observed in Alzheimer's disease [74]. On the other hand, the A30P and E46K variants of α-Syn induced a strong pro-inflammatory response, combined with reduced phagocytic capacity, reflecting a classical activation state, which is clearly associated with the most cytotoxic situation.

Taken together, our results suggest that extracellular, non-aggregated Wt α-Syn produces a moderate to low pro-inflammatory response in glia, together with a reduction of the immunoregulatory response, and a moderate stimulation of Th1 chemokine secretion. The A30P and E46K pathological variants, on the other hand, can induce strong pro-inflammatory and immunoregulatory responses, together with marked increases in chemokine release levels, both in total glia and microglia. This exacerbated native immune response generated by these two α-Syn variants might explain the earlier onset and more rapid evolution of these two genetic forms of PD as compared to the sporadic variety. Intriguingly, our results from the pathologically-linked A53T variant, apart from a weak effect on IL-6 levels, did not provoke a significant native immune response. This finding suggests that there are other mechanisms of neurodegeneration that can contribute to the pathogenesis of PD, perhaps involving adaptive immune responses that might be promoted specifically by the A53T variant.

In comparison to the classical sporadic form of PD, the clinical phenotypes associated with mutations in α-Syn are characterized by an earlier disease onset but a reduced prevalence of tremor [75]–[79]. Studies that help to correlate the different α-Syn variants with the mechanism of neurodegeneration, and ultimately with disease progression, are therefore of considerable importance. However, we should be cautious about preclinical studies in animal models relating to translational research, as immune responses might vary between mice and human systems [80]. Equally important is to mention the lack of physiologically oriented studies employing human primary microglia, rather than immortalised cell lines. In this context, our results on the effects on glial native immunity exerted by extracellular, non-aggregated Wt α-Syn and the various familial PD-linked variants could be of value in the development of rational approaches towards effective control of immune responses that are associated with PD.

## Materials and Methods

### α-synuclein overexpression, purification, and preparation

Human wild-type α-Syn and the A30P, E46K and A53T mutants were overexpressed in *E. coli* BL21(DE3) cells using plasmid pT7-7 and purified as described previously [81] with minor modifications, as follows. After cell transformation, BL21(DE3)-competent cells were grown in LB in the presence of ampicillin (100 µg/ml). Protein expression was induced with 1 mM IPTG, and cells were harvested by centrifugation at 3,500 g after shaking at 37°Cfor 4 hours. The cell pellet was resuspended in 10 mM Tris–HCl (pH 8.0), 1 mM EDTA, and EDTA-free protein inhibitor cocktail (Roche Diagnostics, Burgess Hill, UK), and lysed by multiple freeze–thaw cycles and sonication. The cell suspension was boiled for 20 min and centrifuged at 20,000 g. Streptomycin sulphate was added to the supernatant to a final concentration of 10 mg/ml and the mixture was stirred for 15 min at 4°C. After centrifugation at 13,500 rpm, the supernatant was collected and ammonium sulphate was added (to 0.36 g/ml). The solution was stirred for 30 min at 4°C and centrifuged again at 13,500 rpm. The pellet was resuspended in 25 mM Tris–HCl (pH 7.7), and loaded onto an HQ/M-column on a BioCAD (Applied Biosystems, Foster City, USA) workstation. The synuclein proteins were eluted at ca. 300 mM NaCl with a salt gradient from 0 mM to 600 mM NaCl. The pure α-Syn fractions were pooled together and dialyzed extensively at 4°C against water. Protein purities were >95% as determined by SDS-PAGE, and molecular masses were confirmed by electrospray mass spectrometry.

In order to get rid of any possible bound contaminants, the purified proteins were subjected to denaturation with 6 M urea in 20 mM Tris/HCl (pH 8.0) for 1 hour at room temperature and subsequently dialyzed at 4°C against 20 mM Tris/HCl (pH 8.0) containing 3 M, 1.5 M, 0.75 M, and 0.375 M urea, and finally against water. Afterwards, the protein preparations were concentrated by centrifugation using a 10 k Amicon Ultra-Centrifugal Filter Device (Millipore Iberica, Madrid, Spain), and passed under sterile conditions through a 0.22-µm filter, and stored at −80°C.

### PAGE electrophoresis analyses of the α-Syn preparations

Ten µg of protein were loaded onto a 15% SDS-PAGE gel (the loading buffer containing 0.1 M DTT), or 25 µg onto a 4–12% gradient Tris-glycine native PAGE gel (Lonza Group, Basel, Switzerland), and subjected to electrophoresis at 200 V. Gels were stained with Blue Silver [82], and destained with water. For Blue-Native PAGE (BN-PAGE), samples were prepared according to the protocol described previously for purified samples [83] with some modifications. Briefly, 12 µg of protein for experimental samples and 20 µg for protein markers were loaded on a continuous 15% poly-acrylamide gel. Ovalbumin and bovine serum albumin (Sigma-Aldrich, St. Louis, USA) were used as protein markers for native PAGE and BN-PAGE assays. After electrophoresis, gels were destained in a solution of 50% methanol and 10% acetic acid until the bands were clearly visible.

### Time-course determination of total α-Syn and oligomer content in the medium after incubation with cells

For a quantitative assessment of the oligomeric content of culture supernatants, a specific sandwich ELISA assay [60] and a calibration curve with oligomers of an α-Syn variant carrying six point mutations (V63A, T64S, N65H, V66L, V71F, T72S), were used. Such oligomers were prepared as follows: a 700 µM solution in PBS of the mutant α-Syn was incubated overnight at RT, and subjected to centrifugation with a 100 kDa cut-off centrifugal filter (Millipore #UFC510024). The resulting retentate was rinsed with 150 µl of cold PBS and subjected to centrifugation as before. This procedure was repeated three more times, in order to eliminate the non-oligomerized protein. The oligomeric nature of the prepared α-Syn fraction was confirmed by native PAGE and Western blot (not shown), and the α-synuclein protomer concentration was determined with the BCA assay kit (Pierce, Rockford, USA.).

Quantification of total α-Syn species in culture supernatants after 0, 1, 6 and 20 hours of incubation with cells at 37°C was performed by ELISA. Briefly: individual wells of 96-well ELISA plates were coated with 100 µl of cell-free culture supernatants. Plates were incubated at 37°C for 2 hours and washed with 0.5% Tween20/PBS, pH 7.2 (PBST). Plates were blocked with 200 µl of 1% BSA in PBS and incubation at 37°C for 1 hour. After washing with PBST, 100 µl/well of a 1 µg/ml biotinylated anti-α-Syn 211 mouse mAb [Santa Cruz Biotechnology, Santa Cruz, USA; biotinylated as previously described [60] diluted in 1% BSA in PBS were added and plates were incubated at 37°C for 1 hour. After washing with PBST, 100 µl/well of 1∶5000 dilution of ExtrAvidin-alkaline phosphatase (Sigma-Aldrich, St. Louis, USA) in 1% BSA in PBS were added, and plates were incubated at 37°C for 30 min. After washing with PST, 100 µl/well of pNPP substrate (Sigma, St. Louis, USA) were added, and absorbance at 405 nm was measured within 30 minutes.

### Cell cultures

Mixed glial cultures were prepared from the cerebral cortices of 1–3 days-old C57BL/6 mice (University of Seville Animal Core Facility, Seville, Spain) according to previously described methods [33] with some modifications. After mechanical, trypsin-mediated (BioWhittaker, Verviers, Belgium) dissociation, followed by filtration in DMEM-F12 with 10% inactivated FBS (BioWhittaker, Verviers, Belgium), cells were cultured at 37°C onto 12-well plates treated with poly-D-lysine (Sigma-Aldrich, St. Louis, USA). After 2 days, half of the volume of culture medium was carefully changed, and completely changed after 4 days of culture. Cells were used for stimulation or microglial isolation at day 18–22 of culture.

Microglial isolation was carried out according to previously described methods [84] with some modifications. The supernatant was removed and the wells were washed with DMEM-F12 without inactivated FBS; this conditioned medium was stored to be used later. Cells were incubated for 30–45 min with DMEM-F12/trypsin 0.25% solution at 37°C and complete medium was added to inactivate trypsin and the supernatant containing microglial cells collected. Conditioned medium was added to attached microglia; the following day the medium was changed for normal medium, and microglial cultures were stimulated after 5 days post isolation.

### Immunofluorescence analysis

Purified microglia were characterized by immunocytochemistry on the basis of their expression of the pan haematopoietic marker CD45 and of the monocyte/macrophage markers CD11b, F4/80 and CD68. Additionally, the absence of Glial Fibrillary Acidic Protein (GFAP)-positive astrocytes in purified microglial cell cultures was also established. By contrast, trypsin-detached cells, largely astrocytes but containing some microglial cells, were plated back into 12-multiwell tissue culture plates and immunocytochemically processed as mentioned above.

For detection of CD45, CD11b and CD68 cell-surface antigens, cells were fixed for 10 min at room temperature (RT) in PBS containing 4% paraformaldehyde, washed twice in PBS and finally blocked overnight at 4°C in PBS containing 3% bovine serum albumin (BSA). The cells were then incubated for 1 hour at 4°C with a fluorescein isothiocyanate (FITC)-conjugated rat anti-mouse CD45 monoclonal antibody (Leukocyte Common Antigen, Ly-5, clone 30-F11, BD Pharmingen, Franklin Lakes, USA) and a FITC-conjugated rat anti-mouse CD11b monoclonal antibody (clone M1/70, BD Pharmingen, Franklin Lakes, USA). Cells were exposed to primary rat anti-mouse F4/80 monoclonal antibodies (clone Cl:A3-1, Serotec, Oxford, UK) for 1 hour at RT at a final dilution of 1∶100 in PBS. For detection of CD68 and GFAP intracellular antigens, 4% PFA-fixed cells were permeabilized and blocked overnight at 4°C in PBS containing 3% BSA and 0.5% of the permeabilizing detergent Triton-X-100. The following day, cells were incubated for 1 hour at 4°C in PBS containing a dilution of 1∶100 rat anti-mouse monoclonal CD68 antibodies (clone FA-11, Serotec, Oxford, UK) and a 1∶300 dilution of anti-GFAP mouse monoclonal antibodies (clone G-A-5, Sigma-Aldrich, Saint Louis, USA). Labelling with F4/80 and CD68 primary antibodies was detected from fluorescence measurements after incubating cells for 30 min at RT in PBS containing 1∶100 diluted FITC-conjugated goat anti-rat IgG secondary antibodies (Jackson ImmunoResearch, West Grove, USA). For GFAP detection, an Alexa Fluor 488 nm goat anti-mouse IgG secondary antibody solution (Invitrogen, Paisley, UK) was added for 30 min at RT at a final dilution of 1∶300 in PBS. Immunofluorescence images were captured with an inverted fluorescence microscope Olympus IX71 using the digital image processing softwares DP Controller and DP Manager (Olympus Europa, Hamburg, Germany).

### Phagocytosis assays

Fluoresbrite^TM^ carboxylate 0.75 µ microspheres (2.64% Solid-Latex; Polysciences Inc, Warrington, USA) were used as fluorescein-conjugated tracker microparticles for measuring the phagocytosis capacity of differentially activated microglial cells. One hour before starting the phagocytosis assay, fluorescent microspheres (1.08×10^11^ particles/ml) were mixed at a ratio of 1 µl/20 µl inactivated FBS (BioWhittaker, Verviers, Belgium) and incubated for 1 hour at 37°C in order to opsonise fully the carboxylate groups. The mixture of microspheres and FBS was then resuspended in fresh DMEM-F12 medium (BioWhittaker, Verviers, Belgium), with L-glutamine and P/S antibiotics supplements to obtain normal 10% FBS-supplemented media containing 5.4×10^8^ microspheres/ml.

After removal of 400 µl of supernantant from the α-Syn-stimulated microglial cell cultures, a volume of 150 µl of resuspended microspheres was added to each well to obtain a final concentration of 1.08×10^8^ particles/ml. The particles were then homogenously distributed throughout each well by gentle movements of the plate and incubated for 1 hour at 37°C. The medium containing non-phagocytosed microspheres was then removed and the cells were washed with PBS prior to their fixation with 4% paraformaldehyde in PBS for 30 min at 4°C. One ml of PBS containing the nuclear fluorescent dye Hoechst 33342 (1 µg/ml) was then added to the cells, and the plates were stored at 4°C for a minimum of 24 hours before being analyzed.

### Determination of the phagocytic index

For each cell culture condition, the phagocytic capacity of microglial cells was determined by analysing fluorescent images of phagocytosed FITC-labelled microspheres and by staining cell nuclei with Hoechst 33342. An Olympus IX71 fluorescence microscope equipped with the digital image processing softwares DPController and DPManager (Olympus Europa, Hamburg, Germany) was used.

For each random field, a mean phagocytic index was calculated by determining the intensity of specific green and blue fluorescence emissions with digital imaging analysis software MetaMorph (MDS Analytical Technologies, Toronto, Canada). Specific green fluorescence emitted by FITC-microspheres was determined by subtracting the mean background fluorescence calculated from three different areas of the image where no cells were present, from the overall mean green fluorescence of the entire image. Specific blue emitted nuclear fluorescence was then calculated in a similar manner. The phagocytic index corresponds to the specific green/blue ratio. The mean phagocytic index was calculated from 4 random fields of cells (>100 cells) and was considered as a representative value of the phagocytic capacity of the microglial cells, as previously determined for mouse peritoneal macrophages within normal media for 24 hours with 1 µl/ml of lipopolysacharide (LPS).

### qRT-PCR

Expression of GFAP and CD11b was determined by using a two-step quantitative real-time PCR. Total RNA from two microglial culture wells, one without treatment and one treated with LPS for 16 hours, was extracted using the RNeasy Micro Kit (Qiagen GmbH, Hilden, Germany) according to the manufacturer's protocol. One µg of RNA was reverse-transcribed using the Quantitect Reverse Transcription kit (Qiagen GmbH, Hilden, Germany) according to the manufacturer's protocol. qPCR was performed with SYBR® Premix EX TaqTM (Perfect Real Time) (Takara Bio Inc., Otsu, Shiga, Japan) on an ABI Prism 7500 Real Time PCR System. Primers used were: HPRT_For: 5′-GTAATGATCAGTCAACGGGGGAC-3′, HPRT_Rev: 5′-CCAGCAAGCTTGCAACCTTAACCA-3′; GFAP_For: 5′-ATCGAGATCGCCACCTACAG-3′, GFAP_Rev: 5′-CTCACATCACCACGTCCTTG-3′; CD11b_For: 5′-CAGATCAACAATGTGACCGTATGG-3′, CD11b_Rev: 5′-CATCATGTCCTTGTACTGCCGC-3′). Multiple transcripts were analyzed simultaneously for 40 cycles using an optimized qRT-PCR thermal profile. Changes in gene expression were determined using the 2^−ΔΔCt^ method with normalization to endogenous hypoxanthinephophoribosyltransferase (HPRT) control.

### Cytotoxicity assays

The cytotoxic effect of the α-Syn variants under study was evaluated from the extent of LDH release with by using the LDH Cytoxicity Detection Kit (Roche, Basel, Switzerland) in MGC, following stimulation with the maximum concentration of wild-type α-Syn, its mutants or LPS (Sigma-Aldrich, St. Louis, USA), used in the present experiments (5 µg/ml for α-synuclein samples and 1 µg/ml for LPS). The limiting value in each case was determined using 6 µM MG132 (Sigma-Aldrich, St. Louis, USA), which is known to be lethal for cells at the concentration used, again using the manufacturer's protocol.

### Cytokine release measurements

Glial mixed cultures and isolated microglial cultures were stimulated with Wt α-Syn and its mutational variants at different concentrations (5 µg/ml and 1 µg/ml for MGC and 1 µg/ml and 0.2 µg/ml for MiG) for 20 hours. LPS at a concentration of 1 µg/ml, and culture medium alone were used as positive and negative controls, respectively. Culture supernatants were harvested and centrifuged at 700 g for 5 min. and cell-cleared supernatants were recovered and stored at −80°C before cytokine measurement. IL-6, IL-1β, TNF-α, IFN-γ and IL-10 levels were assayed using Mouse IL-6/IL-1β/TNF-α/IFN-γ/IL-10 BD OptEIA ELISA set (BD Biosciences, Madrid, Spain) according to the manufacturer's protocol. Chemokine levels in the culture supernatants were determined by a specific sandwich ELISA by using capture/biotinylated detection antibodies obtained from Peprotech (London, UK) according to the manufacturer's recommendations. Cytokine profiles shown are representative of three independent experiments.

### Ethics statement

All animals were handled in strict accordance with good animal practice as defined by the relevant national/EU guidelines and the CEA-CABIMER Experimental Animal Committee, and all animal work was approved by the appropriate committee (file CEA-2010-14).

### Data analysis

All values are expressed as mean ± S.E.M. Statistical significance (Student's test, two-tailed) was evaluated using SPSS Statistics 17.0 (IBM Company, Chicago, USA).

## Supporting Information

Figure S1 Time-course quantitation of total α-Syn in cell culture supernatants. Total α-Syn content measured by direct ELISA in culture supernatants recovered after addition of wild-type α-Syn to mixed glial cultures and incubation for 0, 1, 6 and 20 hours at 37°C.
(0.06 MB TIF)

Figure S2 TNF-α and IFN-γ release profile of α-Syn-stimulated primary microglial cultures. TNF-α (A) and IFN-γ (B) levels were measured by ELISA in culture supernantants of microglia after a 20-hour treatment with exogeneously added α-Syn variants, or lipopolysaccharide (LPS). Values are mean ± S.E.M. (n = 3). The results shown are representative of two independent experiments.
(0.13 MB TIF)
