# Supplementary figures and images for: Glial Innate Immunity Generated by Non-Aggregated Alpha-Synuclein in Mouse: Differences between Wild-type and Parkinson's Disease-Linked Mutants
Source: PLoS One. 2010 Oct 26;5(10):e13481. doi: 10.1371/journal.pone.0013481 (PMC2964342; doi:10.1371/journal.pone.0013481)

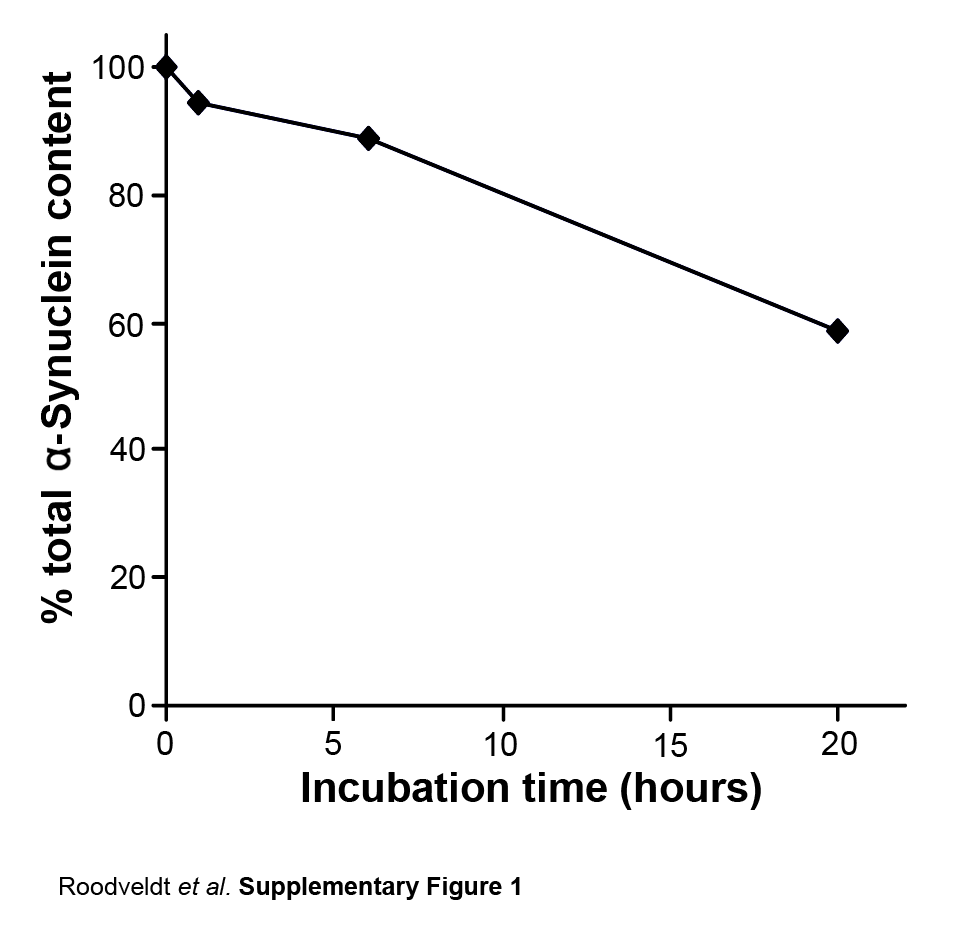

Supplement: Figure S1 — Time-course quantitation of total α-Syn in cell culture supernatants. Total α-Syn content measured by direct ELISA in culture supernatants recovered after addition of wild-type α-Syn to mixed glial cultures and incubation for 0, 1, 6 and 20 hours at 37°C. (0.06 MB TIF) [file pone.0013481.s001.tif]

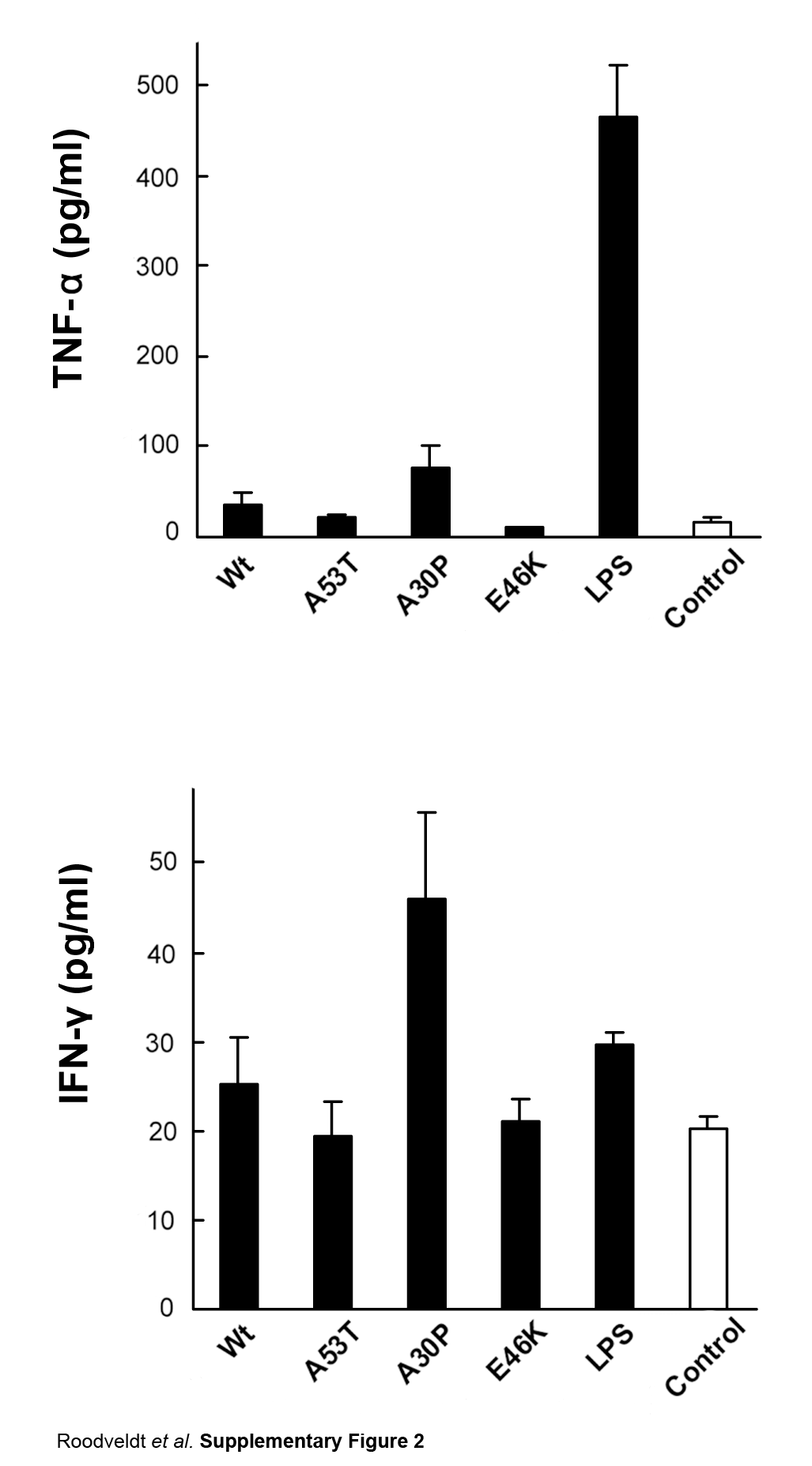

Supplement: Figure S2 — TNF-α (A) and IFN-γ (B) levels were measured by ELISA in culture supernantants of microglia after a 20-hour treatment with exogeneously added α-Syn variants, or lipopolysaccharide (LPS). Values are mean ± S.E.M. (n = 3). The results shown are representative of two independent experiments. (0.13 MB TIF) [file pone.0013481.s002.tif]
